# Impact of pelvicalyceal anatomical variation on surgical outcomes of endoscopic combined intrarenal surgery

**DOI:** 10.1002/bco2.209

**Published:** 2022-12-16

**Authors:** Kengo Kawase, Shuzo Hamamoto, Kazumi Taguchi, Takaaki Inoue, Shinsuke Okada, Teruaki Sugino, Masahiko Isogai, Koei Torii, Takahiro Yanase, Tomoki Okada, Tatsuya Hattori, Ryosuke Chaya, Atsushi Okada, Takahiro Yasui, the SMART Study Group

**Affiliations:** ^1^ Department of Nephro‐Urology Nagoya City University Graduate School of Medical Sciences Nagoya Japan; ^2^ SMART Study Group Japan; ^3^ Department of Urology Hara Genitourinary Hospital Kobe Japan; ^4^ Department of Urology Gyotoku General Hospital Ichikawa Japan

**Keywords:** endoscopic combined intrarenal surgery, multitract percutaneous nephrolithotomy, pelvicalyceal anatomy, renal calculi, upper pole calyceal stone

## Abstract

**Objectives:**

The objective of this work is to investigate the impact of the pelvicalyceal anatomical system (PCS) on calyceal stone formation and surgical outcomes of endoscopic combined intrarenal surgery (ECIRS) for renal and/or proximal ureteral stones with a diameter >15 mm.

**Patients and methods:**

PCS was classified as Type I (single pelvis) or Type II (divided pelvis) according to the simple anatomical Takazawa classification. Using prospectively collected data from January 2016 to April 2020, 219 patients were retrospectively reviewed. After excluding patients who underwent a staged procedure, had hydronephrosis greater than grade 2, prior nephrostomy tubes, and failed to access the renal collecting system, 115 patients (Type I: 81, Type II: 34) were included, and the distribution of calyceal stones and surgical outcomes in ECIRS were compared between Types I and II PCS.

**Results:**

The median number of renal stone calyces in the Type II group was significantly more than that in the Type I group (*p* = 0.016). In particular, the Type II group possessed more upper stone calyces. Multivariate logistic regression analysis revealed that Type II PCS was associated with an increased odds ratio (OR) for the presence of upper stone calyces (OR: 2.93, *p* = 0.018). The stone‐free (SF) status at 1 month after surgery, confirmed by abdominal plain radiography, was significantly higher in the Type I group compared with that in Type II (67.9% vs. 39.4%, respectively; *p* = 0.006). The requirement for additional surgical interventions was significantly higher in the Type II group compared with that in Type I (35.4% vs. 7.4%, respectively; *p* < 0.001). Multivariate analysis revealed that the number of stone calyces (OR: 4.26; *p* = 0.001) and Type II PCS (OR: 3.43; *p* = 0.009) were independent predictors of residual stones after ECIRS.

**Conclusion:**

We first revealed that the anatomic properties of PCS play a role in both upper calyceal stone formation and in the success of the ECIRS procedure. Because the SF rate in Type II PCS was significantly lower than that in Type I PCS, additional percutaneous nephrolithotomy tracts might be required, even for ECIRS.

## 1 INTRODUCTION

Current surgical modalities for renal stones include percutaneous nephrolithotomy (PCNL), retrograde intrarenal surgery (RIRS) using flexible ureteroscopy (fURS), and extracorporeal shock wave lithotripsy. PCNL is the preferred treatment for renal stones >2 cm.^1^ However, PCNL requires a large nephrostomy tract or multiple access tracts for complete stone clearance. The rigid nephoscope cannot access all calyces through a single sheath; hence, multitracts are required in as many as 20–58% of PCNL. This might be associated with severe renal parenchymal damage and intrarenal bleeding.^2^, ^3^

In contrast, endoscopic combined intrarenal surgery (ECIRS) has been developed as a hybrid therapy with simultaneous PCNL and RIRS.^4^ The flexibility of fURS contributes to treating residual fragments that often reside in calyces and cannot be reached using a rigid nephroscope. Therefore, ECIRS could avoid multiple access tracts and decrease the associated morbidity while achieving complete stone clearance.^5^, ^6^, ^7^

**FIGURE 1:**
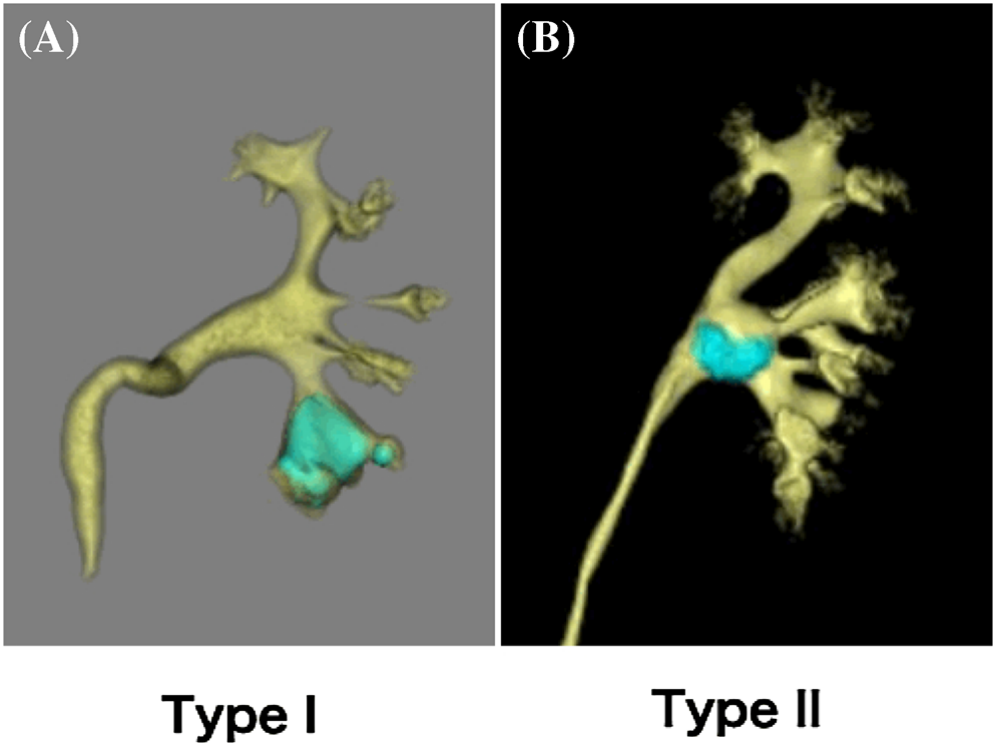
Takazawa classification of the pelvicalyceal anatomical system of representative images of CT urography. (A) Type I, (B) Type II

Predictive factors for residual fragments after PCNL have been reported, including large stone burden, complete staghorn stones, presence of secondary calyceal stones, and body mass index (BMI).^8^, ^9^ However, the anatomical properties of the pelvicalyceal system (PCS) may also influence PCNL outcomes. Yazici et al. reported that PCS Type B1, which was proposed by Sampaio,^10^ causes difficulty with renal calculi within itself from other calyceal accesses and requires multiple tracts for complete stone clearance.^11^ However, to date, the effects of PCS anatomy on ECIRS outcomes have not been evaluated. Currently, the Sampaio classification is the most common anatomical classification for PCS. It is classified into four PCS types according to drainage from the polar regions and the mid‐zone. However, it is difficult to intuitively understand and not practical for endoscopic intrarenal surgery. In 2018, Takazawa et al. developed a new simple classification for the renal pelvis that was useful for endoscopic surgery based on the analysis of multiple CT‐urography findings.^12^ They classified the morphology of the pelvis into Type I (single pelvis) and Type II (divided pelvis), according to the branch pattern of the renal pelvis. Briefly, in Type I, the pelvis forms a true pelvis that is not bifurcated; on the other hand, in Type II the pelvis is bifurcated into the upper and lower branches with the bifurcation point between the upper and middle calyces (Figure 1). In this study, we aimed to investigate the impact of clinical differences in PCS anatomy between Types I and II on surgical outcomes of ECIRS.

## 2 PATIENTS AND METHODS

### 2.1 Patient population

This study used prospectively collected data evaluating the outcomes of ECIRS for renal and/or proximal ureteral stones at a single centre at Nagoya City University Hospital from January 2016 to April 2020. We retrospectively collected data from 219 patients from our prospective dataset and electronic medical records under the approval of the Institutional Review Board of Nagoya City University Graduate School of Medical Sciences (60‐19‐0083), and informed consent was obtained using an opt‐out model. All patients for whom ECIRS was considered primary surgery according to the European Association of Urology and the American Urological Association guidelines were eligible for this study. The indications of stone characteristics were the detection of renal and/or proximal ureteral stones with a diameter >15 mm requiring ECIRS. Patients who underwent staged procedures, had hydronephrosis (greater than grade 2 according to the Ellenbogen classification^13^), prior nephrostomy tubes, and failed to access the renal collecting system either percutaneously or retrogradely were excluded from the analysis. A total of 115 patients were included in this study.

### 2.2 Data collection

Preoperative patient evaluations included age, BMI, Eastern Cooperative Oncology Group Performance Status (ECOG PS), clinical examination, and routine laboratory tests. Clinical and imaging examinations (ultrasound, computed tomography [CT], and abdominal plain radiography [KUB]) were performed to evaluate the PCS anatomy, stone volume, stone calyces, and location of the calculi, including staghorn stone. The PCS of the patient was classified using preoperative enhanced CT as Type I or Type II according to the Takazawa classification.^12^ Stone calyces were defined as the renal calyces with stones.

The primary outcomes of this study were to evaluate the stone‐free (SF) rate and additional stone treatment after the procedure. The SF status was determined as follows: No residual calculi >4 mm detected by KUB 1 day or month after ECIRS, and no residual calculi >2 mm by CT 3 months after the procedure. The secondary outcomes were total surgical duration, number of tracts, and complication rates. Stone volume was calculated using the formula described previously by Tiselius (length × width × 3.14 × 0.25).^14^ The surgical duration was recorded from the initial insertion of a cystoscope to the final ureteral stent placement. Temperature >38.0°C for at least 1 day was defined as postoperative fever. All patients were evaluated for any perioperative complications according to the modified Clavien classification.^15^

### 2.3 Surgical technique

Preoperative urine cultures were performed, and antibiotics were changed based on their results. The ECIRS technique was similar to previously reported methods.^16^, ^17^ All ECIRS procedures were performed in the prone split‐leg (PSL) or Galdakao‐modified supine Valdivia (GMSV) positions throughout the operation, at the surgeon's discretion considering stone and anatomical features.

Two urologists worked simultaneously to fragment the renal calculi: One performed PCNL and the other performed RIRS. A ureteral access sheath (10/12F; Bi‐Flex, Rocamed, Monaco, Italy) was placed to facilitate frequent insertion of fURS (URF‐V2; Olympus Corporation, Tokyo, Japan). Percutaneous renal access was performed using ultrasonography with or without fURS monitoring. The selection of the puncture calyx was at the discretion of the PCNL surgeon for efficient treatment. Thereafter, the percutaneous operating sheath was inserted using a one‐step dilator. We utilized a Holmium‐YAG laser (Cyber Ho; Rocamed, Monaco, Italy) through a fURS (common laser settings used were 0.5–1.0 J at 5–10 Hz) and a pneumatic lithotripsy (Swiss LithoClast® Master J; Electro Medical Systems, Nyon, Switzerland) through a mini‐perc (Karl Storz, Tuttlingen, Germany). Operating sheath sizes (17.5F, 21F, or 24F) were selected depending on the stone's complexity. The stone burdens were crashed into tiny fragments and washed out through the PCNL sheath using retrograde automated irrigation (UROMAT E.A.S.I.®; Karl Storz). At the end of each surgery, a 4.8F ureteral stent tube and 14F urinary catheter were routinely placed for 1–4 weeks and 1 day, respectively. A 12Fr nephrostomy tube was used in cases of risk of postoperative infection or bleeding.

### 2.4 Statistical analysis

All data were statistically analysed by two physicians (KK, SH) using Easy R (EZR) for R.^18^ The quantitative variables were expressed as means ± standard deviations in a normal distribution or expressed as the median (interquartile ranges) in the case of a non‐normal distribution. Categorical data are expressed as numbers (percentages). Student's *t* tests or Mann–Whitney *U* tests were used to compare the quantitative data, whereas Fisher's exact and chi‐square tests were used to compare the categorical variables. The predictive factors of residual fragments status and additional surgery were evaluated using a logistic regression model by adjusting the covariates assumed to impact these outcomes at the time of surgical planning. The cut‐off values for stone volume and maximum stone density were determined to be median. A *p* value <0.05 was considered to indicate statistical significance. In subgroup analyses, the factors associated with the presence of upper calyceal stones were evaluated using a logistic regression model.

## 3 RESULTS

### 3.1 Patient and stone characteristics

The patient and stone characteristics are summarized in Table 1. In total, 115 patients (Type I: 81, Type II: 34) were included in this study. The mean age at surgery was 55.9 and 56.7 in the Type I and Type II groups, respectively. On the left side, there were 55.6% (Type I) and 76.5% (Type II) stones, respectively, and the difference was significant (*p* = 0.038). The median number of stone calyces in the Type II group was significantly greater than that in the Type I group (*p* = 0.016); especially upper pole stone calyces were greater in the Type II group. The ratio with superior calyceal stone were significantly higher in the in the Type II group than that that in the Type I group (*p* = 0.024). The other preoperative background characteristics of the two groups were comparable.

**TABLE 1:** Patient and stone characteristics of Type I and Type II groups

		Type I	Type II	*p* value
		*n* = 81	*n* = 34	
Age	Years	55.9 (16.6)	56.7 (13.5)	0.813
Female sex (%)		36 (44.4)	12 (35.3)	0.834
BMI	kg/m^2^	23.95 (4.78)	24.72 (3.46)	0.386
ECOG PS	0	79 (96.3)	33 (94.3)	0.618
	1	1 (1.2)	0 (0.0)	
	2	1 (1.2)	0 (0.0)	
	>3	1 (1.2)	2 (5.7)	
Left side (%)		45 (55.6)	26 (76.5)	0.038
Prestenting (%)		9 (11.1)	3 (8.8)	1
Hydronephrosis (%)	Grade 0	59 (72.0)	27 (79.4)	0.489
	Grade 1	23 (28.0)	7 (20.6)	
Number of stone calyces				
All calyceal stones		2 [1, 3]	3 [1, 6]	0.016
Top pole		0 [0, 0]	0 [0, 1]	0.020
Upper pole		0 [0, 0]	0 [0, 1]	0.003
Middle pole		0 [0, 1]	1 [0, 2]	0.109
Lower pole		1 [0, 1]	1 [0, 2]	0.108
Bottom pole		1 [0, 1]	1 [0, 1]	0.223
Presence of superior calyceal stones		18 (22.2)	15 (44.1)	0.024
Staghorn (%)		45 (55.6)	17(50.0)	0.064
Total stone volume	cm^3^	6.11[3.22, 13.86]	9.45[2.90, 25.19]	0.423
Maximum stone density	HU	1287 [979, 1479]	1145 [755, 1486]	0.314
Stone analysis	CaOx/CaP	66 (81.5)	25 (73.5)	0.561
	UA	5 (6.2)	4 (11.8)	
	Cystine	3 (3.7)	3 (8.8)	
	Struvite	6 (7.4)	2 (5.9)	
	Others	1 (1.2)	0 (0.0)	

*Note*: Mean (SD). Median [25% IQR, 75% IQR], number (%).

Abbreviations: BMI, body mass index; HU, Hounsfield unit.

### 3.2 Intraoperative parameters and surgical outcomes

Comparisons of intraoperative parameters and surgical outcomes are summarized in Table 2. The surgical position did not differ between the two groups. The ratio of upper pole punctures was higher in the Type II group compared with that of the Type I group (17.6% vs. 2.5%, respectively). The trends in the selected puncture calyx during surgery were significantly different (*p* = 0.019).

**TABLE 2:** Comparison of intraoperative parameters and surgical outcomes

		Type I	Type II	*p* value
		*n* = 81	*n* = 34	
Surgical positioning	PSL	61 (75.3)	25 (73.5)	0.819
	GMSV	20 (24.7)	9 (26.5)	
Puncture calyx (%)	Upper	2 (2.5)	6 (17.6)	0.019
	Middle	25 (30.9)	10 (29.4)	
	Lower	54 (66.6)	18 (53.0)	
Tract size	12Fr	2 (2.4)	0 (0)	0.494
	17.5Fr	74 (91.4)	30 (88.2)	
	21Fr >=	5 (6.2)	4 (11.8)	
Tract number	Single	77 (95.1)	33 (97.1)	1
	Double	4 (4.9)	1 (2.9)	
Tubeless (%)		63 (77.8)	21 (61.8)	0.106
Surgical duration	Minutes	119.7 (42.9)	124.8 (50.1)	0.583
Stone‐free status^a^ 1 day after surgery		56 (70.0)	13 (39.4)	0.003
Stone‐free status^b^ 1 month after surgery		55 (67.9)	13 (39.4)	0.006
Stone‐free status^c^ 3 months after surgery		59 (72.8)	18 (52.9)	0.051
Additional surgical intervention		6 (7.4)	12 (35.3)	<0.001
Complications^d^ within 3 months after surgery	1	5 (6.2)	3 (8.8)	0.340
	2	21 (25.9)	6 (17.6)	
	3	0 (0.0)	1 (2.9)	
Postop fever^e^		18 (23.1)	6 (17.6)	0.621
Serum Hb decrease 1 day after surgery	g/dL	1.40 [0.70, 2.00]	1.35 [0.10, 1.97]	0.394

^a^ Stone‐free status was defined as <4 mm in size by KUB.

^b^ Stone‐free status was defined as <4 mm in size by KUB.

^c^ Stone‐free status was defined as <2 mm in size by CT.

^d^ Complications were shown as Clavien–Dindo classification.

^e^ Defined as greater than 38.5°C mean (SD). Median [25% IQR, 75% IQR], number (%).

Abbreviations: GMSV, Galdakao‐modified supine Valdivia; Hb, haemoglobin; PSL, prone split‐leg.

As the primary outcome, the SF status at 3 months after surgery by CT was 72.8% and 52.9% in the Type I and Type II groups, respectively (*p* = 0.051), whereas the SF status at 1 month after surgery, confirmed by KUB radiographs, was significantly higher in Type I than in Type II (67.9% vs. 39.4%, respectively; *p* = 0.006). Significantly higher additional surgical interventions were required in the Type II group compared with that in the Type I group (35.4% vs. 7.4%, respectively; *p* < 0.001). Regarding secondary outcomes, no significant differences between the two groups were observed in surgical duration, tract numbers, and complication rates within 3 months following surgery. There were no significant differences in other intraoperative parameters or specific complications, including the incidence of postoperative fever and a decrease in haemoglobin levels.

### 3.3 Predictive factors for residual stone

Univariate analysis revealed that predictive factors significantly associated with residual stones 1 month after surgery included calyceal stones, staghorn, and Type II pelvicalyceal anatomy. Multivariate logistic regression analysis indicated that the number of stone calyces (OR: 4.26; *p* = 0.001) and Type II pelvicalyceal anatomy (OR: 3.43; *p* = 0.009) were independent predictors of residual stones (Table 3).

**TABLE 3:** Logistic regression analysis of factors associated with surgical outcomes

		Residual stones 1 month after surgery			
		Univariate		Multivariate	
		Odds ratio (95% CI)	*p* value	Odds ratio (95% CI)	*p* value
Age	>55 years	1.35 (0.63–2.90)	0.450		
BMI	>24 kg/m^2^	1.56 (0.73–3.32)	0.250		
Number of stone calyces	>2	5.49 (2.42–12.4)	<0.001	4.26 (1.76–10.3)	0.001
Staghorn		2.62 (1.20–5.71)	0.016	2.14 (0.79–5.79)	0.133
Total stone volume	>7000 mm^3^	2.03 (0.95–4.34)	0.068	0.98 (0.37–2.58)	0.980
Maximum stone density	>1200 HU	1.60 (0.75–3.40)	0.220		
Pelvicalyceal anatomy	Type II	3.25 (1.41–7.54)	0.005	3.43 (1.35–8.72)	0.009

Abbreviations: BMI, body mass index; CI, confidence interval.

### 3.4 Predictive factors associated with the presence of superior calyceal stones

Further evaluation with logistic regression analysis showed that Type II pelvicalyceal anatomy was associated with an increased OR for the presence of superior calyceal stones (OR: 2.93, *p* = 0.018) (Table 4).

**TABLE 4:** Logistic regression analysis of factors associated with superior calyceal stones

		Univariate		Multivariate	
		Odds ratio (95% CI)	*p* value	Odds ratio (95% CI)	*p* value
BMI	>24 kg/m^2^	0.83 (0.37–1.87)	0.66	0.64 (0.27–1.54)	0.323
Female sex		0.61 (0.26–1.45)	0.27	0.54 (0.21–1.34)	0.186
Left side		1.35 (0.58–3.14)	0.49	1.16 (0.47–2.82)	0.746
Pelvicalyceal anatomy	Type II	2.76 (1.17–6.50)	0.02	2.93 (1.20–7.18)	0.018

*Note*: years.

Abbreviations: BMI, body mass index; CI, confidence interval.

## 4 DISCUSSION

This is the first study to evaluate the association between PCS anatomy and surgical outcomes of ECIRS. This retrospective study revealed that the Type II group demonstrated a lower SF rate and higher additional surgical intervention, which could negatively impact the surgical outcomes of ECIRS. Moreover, multivariate logistic regression analysis revealed, for the first time, a relationship between the formation of upper pole calyceal stones and Type II PCS anatomy.

To date, few reports have analysed the etiologic role of pelvicalyceal variations in renal stone formation.^19^, ^20^, ^21^ Although these studies demonstrated that large pelvicalyceal volume or the infundibulopelvic angle (IPA) is a significant risk factor that promotes lower calyceal stone formation,^19^, ^20^ few studies have investigated the association between these anatomic factors and upper calyceal stones formation. Akar et al. demonstrated that pelvicalyceal did not appear to be a risk factor for upper pole stone formation. However, the analysed risk factors were upper pole IPA, pelvicalyceal volume, and infundibular width and length, but not the renal PCS type.^21^ Type II PCS anatomy is characterized by the pelvis bifurcating into the upper and lower branches, and the bifurcation point is located between the upper and middle calyces.^12^ A divided pelvis seems to be a significant risk factor for upper pole stone formation. A specific reason remains unclear, however, the infundibulopelvic width and length of the upper calyx in the Type II PCS seem narrower and longer than those in Type I PCS, because Type II PCS has a bifurcation point between the upper and middle calyces. This morphological feature might cause abnormal urodynamics and result in upper pole stone formation. Therefore, Type II PCS possessed more calyceal stones, and upper pole punctures were required more frequently in Type II PCS than in Type I PCS.

Upper pole puncture has some advantages including direct access to the upper calyceal stones and easy access to many calyces due to better manipulation of the nephoscope along the long axis of the kidney. Amaresh et al. documented that upper pole puncture was more efficacious in achieving a complete SF rate with a shorter surgery time than lower pole puncture.^22^ However, upper pole puncture is usually associated with potential complications such as hydrothorax, pneumothorax, and lung injury. Munver et al. reported that 35% supra‐11th and 10% supra‐12th rib punctures resulted in thoracic complications.^23^ However, no thoracic complications were observed in this study. Percutaneous renal access was performed using ultrasonography and fURS monitoring. Ultrasonography‐guided puncture has been reported to eliminate the risk of inadvertent organ injuries.^24^ We believe that ultrasound‐guided puncture with fURS monitoring might contribute to reducing the possibility of thoracic complications due to improved visibility of the renal calyces and surrounding organs.

In this study, the SF rate of ECIRS at 1 month after surgery in the Type II group was significantly lower than that in the Type I group (39.4% vs. 67.9%, respectively; *p* = 0.006). The independent predictors of residual fragments after one session of ECIRS were calyceal stone number (OR: 4.26) and Type II pelvicalyceal anatomy (OR: 3.73). These results were slightly different from the independent predictors of PCNL, including stone volume, number, location, and staghorn calculi, because ECIRS could compensate for the PCNL disadvantages of limited space for the nephoscope manipulation by using antegrade and retrograde devices simultaneously. Some models can predict SF status after PCNL such as S.T.O.N.E. nephrolithometry score^25^ and GUY's stone score.^26^ The S.T.O.N.E. nephrolithometry score is based on five variables, including stone size, tract length, obstruction, number of involved calyces, and stone density, but did not include the anatomy of PCS. GUY's score is decided according to stone number, stone location, renal anatomy, staghorn status, and presence of the spinal code lesions. In GUY's scoring model, abnormal anatomy was defined as abnormal renal anatomy, abnormal collecting system, or a patient with an ileal conduit; however, the importance of Type II or Type I PCS was not mentioned. We first revealed the relationship between PCS anatomy and surgical outcomes of ECIRS. The effects of pelvicalyceal anatomy on SF status by shock wave lithotripsy (SWL), RIRS, and PCNL have not yet been properly evaluated. Regarding SWL, upper calyceal anatomy has no significant effect on stone clearance for upper calyceal calculi.^27^ However, steep IPA might be a risk factor for residual fragments of lower calyceal stones. According to a systematic review, PCS appeared to be the most important predictor of successful treatment of lower calyceal stones.^28^ Verma et al. documented that the infundibular width, IPA, and pelvicalyceal volume significantly affected the SF status.^29^ On the other hand, Binbay et al. documented that only the pelvicalyceal volume significantly affected the success rate of PCNL, while PCS anatomy did not affect outcomes.^30^ Yazici et al. reported that a divided pelvis, which was defined as Type B1 by the Sampaio classification,^10^ resulted in a lower SR rate and required more PNCL tracts than other PCS types^11^ because the divided pelvis made it difficult to reach the stone from other calyceal tracts and prevented the stone from moving to the renal pelvis during manipulations in PCNL. Although ECIRS was an efficient therapy for large renal stone burdens to avoid multiple accesses and related morbidity, additional PCNL tracts might be required to increase the SF rate in cases of Type II PCS.

In this study, we divided the PCS anatomy into two groups using the Takazawa classification,^12^ and some reports have proposed the classification of pelvicalyceal patterns. In 1954, Graves et al.^31^ defined PCS classification according to the shape of the renal pelvis along with calyceal protrusion. In 1998, Sampaio and Mandarim‐de‐Lacerda^10^ classified the PCS system, which was divided into four types according to the drainage of the hilar zone and polar regions. However, most classifications were not sufficiently widespread because the classifications that were proposed based on two‐dimensional images were not practical for endoscopic surgery. The Takazawa classification was proposed based on the analysis of many three‐dimensional CT urography images and might be useful for planning endoscopic treatment strategies. Therefore, there were significant differences in SF rates and additional surgical interventions between Types I and II PCS anatomies.

This study has certain methodological limitations. First, its small sample size and retrospective design limited the evaluation of the relationship between PCS anatomy and surgical outcomes of ECIRS. However, this is the first report discussing the impact of the PCS type defined by the Takazawa classification on ECIRS outcomes. Second, the frequency of left kidney stones in the Type II group was higher than in the Type I group. The reason has yet to be elucidated but should be in the future using large cohort data. Third, the SF rates in both the Type I and Type II groups were relatively inferior to those in previous reports.^7^, ^32^ Because the cohort in this study did not include patients with greater than grade 2 hydronephrosis, it might be difficult to create an ideal PCNL tract. Finally, the surgical positioning and targeted calyx were selected at the surgeon's discretion; therefore, a selection bias may have been possible. However, a preoperative meeting was held with as many surgeons as possible to determine the treatment strategy.

In conclusion, we first revealed that the anatomic properties of the PCS play a role not only in upper calyceal stone formation but also in the success of the ECIRS procedure. Because the SF rate in Type II PCS was significantly lower than that in Type I PCS, additional PCNL tracts might be required even for ECIRS.

## AUTHOR CONTRIBUTIONS

K.K., S.H., T.I., S.O. and T.Y. designed and directed the study. K.K., I.M. and K.T. analysed most of the data with assistance from S.H. K.T., T.S., T.Y., T.O., T.H., R.C., T.E., K.T., S.I., N.M., Y.S., Y.M., K.O., Y.N., M.A., Y.M., S.N., S.K. and A.O. acquired the data. K.T. conducted the statistical analyses. S.H. and K.K. wrote the manuscript. All authors engaged in critical discussion of the results and provided input regarding the manuscript.

## CONFLICT OF INTERESTS

The authors declare that they have no conflict of interest.

## References

[1] Preminger GM , Tiselius HG , Assimos DG , Alken P , Buck AC , Gallucci M , et al. 2007 guidelines for the management of ureteral calculi. Eur Urol. 2007;52(6):1610–31. 10.1016/j.eururo.2007.09.039 18074433

[2] Ganpule AP , Desai M . Management of the staghorn calculus: multiple‐tract versus single‐tract percutaneous nephrolithotomy. Curr Opin Urol. 2008;18(2):220–3. 10.1097/MOU.0b013e3282f3e6e4 18303548

[3] El‐Nahas AR , Shokeir AA , El‐Assmy AM , Mohsen T , Shoma AM , Eraky I , et al. Post‐percutaneous nephrolithotomy extensive hemorrhage: a study of risk factors. J Urol. 2007;177(2):576–9. 10.1016/j.juro.2006.09.048 17222636

[4] Scoffone CM , Cracco CM , Cossu M , Grande S , Poggio M , Scarpa RM . Endoscopic combined intrarenal surgery in Galdakao‐modified supine Valdivia position: a new standard for percutaneous nephrolithotomy? Eur Urol. 2008;54(6):1393–403. 10.1016/j.eururo.2008.07.073 18715696

[5] Marguet CG , Springhart WP , Tan YH , Patel A , Undre S , Albala DM , et al. Simultaneous combined use of flexible ureteroscopy and percutaneous nephrolithotomy to reduce the number of access tracts in the management of complex renal calculi. BJU Int. 2005;96(7):1097–100. 10.1111/j.1464-410X.2005.05808.x 16225535

[6] Hamamoto S , Yasui T , Okada A , Taguchi K , Kawai N , Ando R , et al. Endoscopic combined intrarenal surgery for large calculi: simultaneous use of flexible ureteroscopy and mini‐percutaneous nephrolithotomy overcomes the disadvantageous of percutaneous nephrolithotomy monotherapy. J Endourol. 2014;28(1):28–33. 10.1089/end.2013.0361 23987470

[7] Kuroda S , Ito H , Sakamaki K , Tabei T , Kawahara T , Terao H , et al. Development and internal validation of a classification system for predicting success rates after endoscopic combined intrarenal surgery in the modified Valdivia position for large renal stones. Urology. 2015;86(4):697–702. 10.1016/j.urology.2015.07.002 26190085

[8] el‐Nahas AR , Eraky I , Shokeir AA , Shoma AM , el‐Assmy AM , el‐Tabey NA , et al. Factors affecting stone‐free rate and complications of percutaneous nephrolithotomy for treatment of staghorn stone. Urology. 2012;79(6):1236–41. 10.1016/j.urology.2012.01.026 22465085

[9] Olbert PJ , Hegele A , Schrader AJ , Scherag A , Hofmann R . Pre‐ and perioperative predictors of short‐term clinical outcomes in patients undergoing percutaneous nephrolitholapaxy. Urol Res. 2007;35(5):225–30. 10.1007/s00240-007-0112-6 17786419

[10] Sampaio FJB , Mandarim‐de‐Lacerda CA . Anatomic classification of the kidney collecting system for endourologic procedures. J Endourol. 1988;2(3):247–51. 10.1089/end.1988.2.247

[11] Yazici O , Binbay M , Akman T , Kezer C , Ozgor F , Yuruk E , et al. Is there a difference in percutaneous nephrolithotomy outcomes among various types of pelvicaliceal system? World J Urol. 2013;31(5):1267–72. 10.1007/s00345-012-0907-0 22810053

[12] Takazawa R , Kitayama S , Uchida Y , Yoshida S , Kohno Y , Tsujii T . Proposal for a simple anatomical classification of the pelvicaliceal system for endoscopic surgery. J Endourol. 2018;32(8):753–8. 10.1089/end.2018.0218 29845879

[13] Ellenbogen PH , Scheible FW , Talner LB , Leopold GR . Sensitivity of gray scale ultrasound in detecting urinary tract obstruction. AJR am J Roentgenol. 1978;130(4):731–3. 10.2214/ajr.130.4.731 416685

[14] Tiselius HG , Andersson A . Stone burden in an average Swedish population of stone formers requiring active stone removal: how can the stone size be estimated in the clinical routine? Eur Urol. 2003;43(3):275–81. 10.1016/S0302-2838(03)00006-X 12600431

[15] de la Rosette JJ , Opondo D , Daels FP , Giusti G , Serrano A , Kandasami SV , et al. Categorisation of complications and validation of the Clavien score for percutaneous nephrolithotomy. Eur Urol. 2012;62(2):246–55. 10.1016/j.eururo.2012.03.055 22487016

[16] Hamamoto S , Yasui T , Okada A , Takeuchi M , Taguchi K , Shibamoto Y , et al. Developments in the technique of endoscopic combined intrarenal surgery in the prone split‐leg position. Urology. 2014;84(3):565–70. 10.1016/j.urology.2014.04.020 24929943

[17] Inoue T , Kinoshita H , Okada S , Hamamoto S , Taguchi M , Murota T , et al. Wideband Doppler ultrasound‐guided mini‐endoscopic combined intrarenal surgery as an effective and safe procedure for management of large renal stones: a preliminary report. Urology. 2016;95:60–6. 10.1016/j.urology.2016.05.038 27235750

[18] Kanda Y . Investigation of the freely available easy‐to‐use software ‘EZR’ for medical statistics. Bone Marrow Transplant. 2013;48(3):452–8. 10.1038/bmt.2012.244 23208313 PMC3590441

[19] Nabi G , Gupta NP , Mandal S , Hemal AK , Dogra PN , Ansari MS . Is infundibuloureteropelvic angle (IUPA) a significant risk factor in formation of inferior calyceal calculi? Eur Urol. 2002;42(6):590–3. 10.1016/S0302-2838(02)00451-7 12477655

[20] Gurocak S , Kupeli B , Acar C , Guneri C , Tan MO , Bozkirli I . Pelvicaliceal anatomical variation between stone bearing and normal contralateral kidneys—does it have an impact on stone formation in pediatric patients with a solitary lower caliceal stone? J Urol. 2006;175(1):270–5; discussion 275. 10.1016/S0022-5347(05)00010-8 16406924

[21] Acar C , Küpeli B , Gürocak S , Alkibay T , Güneri Ç , Özkan S , et al. Is pelvicaliceal anatomy a risk factor for stone formation in patients with solitary upper caliceal stone? Urology. 2006;67(6):1159–63. 10.1016/j.urology.2005.12.025 16750255

[22] Amaresh M , Hegde P , Chawla A , de la Rosette JJMCH , Laguna MP , Kriplani A . Safety and efficacy of superior calyceal access versus inferior calyceal access for pelvic and/or lower calyceal renal calculi‐ a prospective observational comparative study. World J Urol. 2021;39(6):2155–61. 10.1007/s00345-020-03409-3 32865690 PMC8216999

[23] Munver R , Delvecchio FC , Newman GE , Preminger GM . Critical analysis of supracostal access for percutaneous renal surgery. J Urol. 2001;166(4):1242–6. 10.1016/S0022-5347(05)65745-X 11547050

[24] Ng FC , Yam WL , Lim TYB , Teo JK , Ng KK , Lim SK . Ultrasound‐guided percutaneous nephrolithotomy: advantages and limitations. Investig Clin Urol. 2017;58(5):346–52. 10.4111/icu.2017.58.5.346 PMC5577331 28868506

[25] Okhunov Z , Friedlander JI , George AK , Duty BD , Moreira DM , Srinivasan AK , et al. S.T.O.N.E. nephrolithometry: novel surgical classification system for kidney calculi. Urology. 2013;81(6):1154–60. 10.1016/j.urology.2012.10.083 23540858

[26] Thomas K , Smith NC , Hegarty N , Glass JM . The Guy's stone score—grading the complexity of percutaneous nephrolithotomy procedures. Urology. 2011;78(2):277–81. 10.1016/j.urology.2010.12.026 21333334

[27] Küpeli B , Acar C , Gürocak S , Güneri C , Karaoglan U , Bozkirli I . Is stone clearance after shockwave lithotripsy in patients with solitary upper‐caliceal stone influenced by anatomic differences in the pelvicaliceal system? J Endourol. 2007;21(1):18–22. 10.1089/end.2006.0156 17263602

[28] Karim SS , Hanna L , Geraghty R , Somani BK . Role of pelvicalyceal anatomy in the outcomes of retrograde intrarenal surgery (RIRS) for lower pole stones: outcomes with a systematic review of literature. Urolithiasis. 2020;48(3):263–70. 10.1007/s00240-019-01150-0 31372691 PMC7220875

[29] Verma A , Tomar V , Yadav S . Complex multiple renal calculi: stone distribution, pelvicalyceal anatomy and site of puncture as predictors of PCNL outcome. Springerplus. 2016;5(1):1356. 10.1186/s40064-016-3017-4 27588249 PMC4988955

[30] Binbay M , Akman T , Ozgor F , Yazici O , Sari E , Erbin A , et al. Does pelvicaliceal system anatomy affect success of percutaneous nephrolithotomy? Urology. 2011;78(4):733–7. 10.1016/j.urology.2011.03.058 21676442

[31] Graves FT . The anatomy of the intrarenal arteries and its application to segmental resection of the kidney. Br J Surg. 1954;42(172):132–9. 10.1002/bjs.18004217204 13209036

[32] Hamamoto S , Okada S , Inoue T , Taguchi K , Kawase K , Okada T , et al. Comparison of the safety and efficacy between the prone split‐leg and Galdakao‐modified supine Valdivia positions during endoscopic combined intrarenal surgery: A multi‐institutional analysis. Int J Urol. 2021;28(11):1129–35. 10.1111/iju.14655 34342062

